# TiO_2_-Nanofillers Effects on Some Properties of Highly- Impact Resin Using Different Processing Techniques

**DOI:** 10.2174/1874210601812010202

**Published:** 2018-03-26

**Authors:** Hawraa Khalid Aziz

**Affiliations:** Department of Dental Technology, Prosthetic Dental Technology, College of Health and Medical Technology, Middle Technical University (MTU), Baghdad, Iraq

**Keywords:** Acrylic denture base, TiO_2_ nanofillers, Microwave, Impact strength, Thermal conductivity, Color stability

## Abstract

**Background:**

The criteria of conventional curing of polymethyl methacrylate do not match the standard properties of the denture base materials.

**Objectives:**

This research was conducted to investigate the addition of TiO_2_ nano practical on impact strength, thermal conductivity and color stability of acrylic resin cured by microwave in comparison to the conventional cured of heat-polymerized acrylic resin.

**Materials and Methods:**

120 specimens made of high impact acrylic resin were divided into two main groups according to the type of curing (water bath, microwave), then each group was subdivided into two groups according to the addition of 3% TiO_2_ nano-fillers and control group (without the addition of TiO_2_ 0%). Each group was subdivided according to the type of test into 3 groups with 10 specimens for each group. Data were statistically analyzed using Student t-test to detect the significant differences between tested and control groups at significance level (*P*<0.05).

**Results:**

According to curing type methods, the results showed that there was a significant decrease in impact strength of microwaved cured resin, but there was no significant difference in the thermal conductivity and color stability of resin. In addition, by using nanofiller, there was a significant increase in the impact strength and color stability with the addition of 3% TiO_2_ nanofillers, but no significant difference was found in the thermal conductivity of the acrylic resin.

**Conclusion:**

The microwave curing of acrylic resin had no change in the color stability and thermal conductivity in comparison to the water bath, but the impact strength was decreased. The addition of 3% TiO_2_ improved the impact and the color stability, but the thermal conductivity did not change.

## 1 INTRODUCTION

The substance which is employed broadly for dentures manufacturing is the PMMA resin, because of its characteristics such as simple laboratory using, low weight, cheap material, good stability in oral condition, low toxicity and appropriate aesthetic and color matching ability [1]. In order to get better physical properties and to simplify the technique, new resins and processing methods have been introduced as an alternative to conventional water-bath [2]. Several types of modified poly-methyl methacrylate have been introduced for denture base construction. These include self-cured resins, high impact strength resins, pour type resins, microwave-cured resins and light-cured resins [3].

Acrylic resin is traditionally cured with a water bath method. Microwave energy processing was first reported as an alternative technique [4]. Processing with dry heat, steam and infra-red induction or dielectric heating has also been used. Microwaves are important additions to this list. The American dental association specification indicated that microwave energy used for curing the acrylic resin further withstands to mechanical foundering than conventionally polymerized acrylic materials [5]. In comparison, the polymerization of heat-cured PMMA is usually carried out in a temperature-controlled water bath for at least 9 hours, while the processing by microwave reduced the period to just three minutes, resulting in the resin with the same finesse as those cured by water bath [6]. The method is fast and clean, and the final resulting product has better accuracy of fit, with a better adaptation of denture base [7].

On the other hand, there is a drawback of the use of PMMA as a denture base material with a low impact strength that leads to the fracture of the denture commonly. So the increase of the impact strength is important to get rid of the denture fracture as a result of its fall accidentally [8, 9]. In addition, declined thermal conductivity of the polymer will have an effect on the wearers’ palatability and taste rating [10].

On the other hand, the huge advancement and development of nanotechnology caused the nano-fillers to reinforce the denture materials and improve mechanical and physical properties of the polymer [11, 12]. Polymer nanocomposites are commonly defined as the combination of a polymer matrix and some particulate additives that have at least one dimension in the nanometer scale [13].Therefore, several attempts were made to change and improve the strength, thermal properties, and hardness of PMMA. These attempts included the addition of filler particles such as zirconia, glass fiber, alumina, tin and copper to resin [14], which depend on the incorporated nano-particles type, size, and shape [15]. There is high interest in using TiO_2_ because of its so many applications, for example as chemical sensor and as a biomedical material. Nano-sized structured TiO_2_ has proved to have antimicrobial properties [16]. Moreover, it is a biocompatible material, cheap with chemical stability and free toxicity, and the two most useful advantages of TiO_2_ are resistance to corrosion and highest strength [17]. It acts as a photo-catalyst that has been used extensively for killing different groups of microorganisms including bacteria, fungi, and viruses [18]. Many studies conduct the use of TiO_2_ in different percentages such as 1% of TiO_2_ nanoparticles which may be incorporated into color-modified acrylic resin powder to enhance its tensile and impact strength, given that they have no adverse effect on other properties [19].

Further researches revealed that the addition of 3% TiO_2_ nanoparticles to heat cured resin improved the impact strength, transverse strength and surface hardness of acrylic resin and decreased water sorption and solubility [20].

Chen *et al*., 2017 revealed that the use of nano-sized 3% TiO_2_ had antibacterial activities compared to the control and blank groups with no significant differences in the cytotoxicity and found no significant differences in the mechanical properties compared to the control group within the range of an appropriate ratio [21]. The different percentages of superinducing TiO_2_ nanofiller 1% and 5% were added to the acrylic which showed that 5% of TiO_2_ nanoparticles can adversely affect its flexural strength [22].

Therefore, this study is oriented to evaluate the effect type of curing methods water bath and microwaved cured on the impact strength, thermal conductivity, and color stability of high impact acrylic resin and evaluate the addition of 3% TiO_2_ on the same properties.

## 2 MATERIALS AND METHODS

A total of 120 specimens were prepared from heat-cured denture base resin. High impact acrylic (Vertex-Dental, Netherlands) was divided into two groups according to the processing technique: Group I: water bath, and Group II: microwave curing. Each group was divided into 2 subgroups: the control group with non-additive filler and the experimental group treated with 3% TiO_2_ Nano-fillers (Nanoshel, Stock no. NS6130-03-352, USA), TiO_2_ nanopowder of 30 nm particle size according to the recommendation of manufacturer instruction. The samples of each group were subdivided depending on the tests (Impact test, thermal conductivity, and color stability test) (N=10).

### 2.1 Sample Preparation

**Fig. (1):**
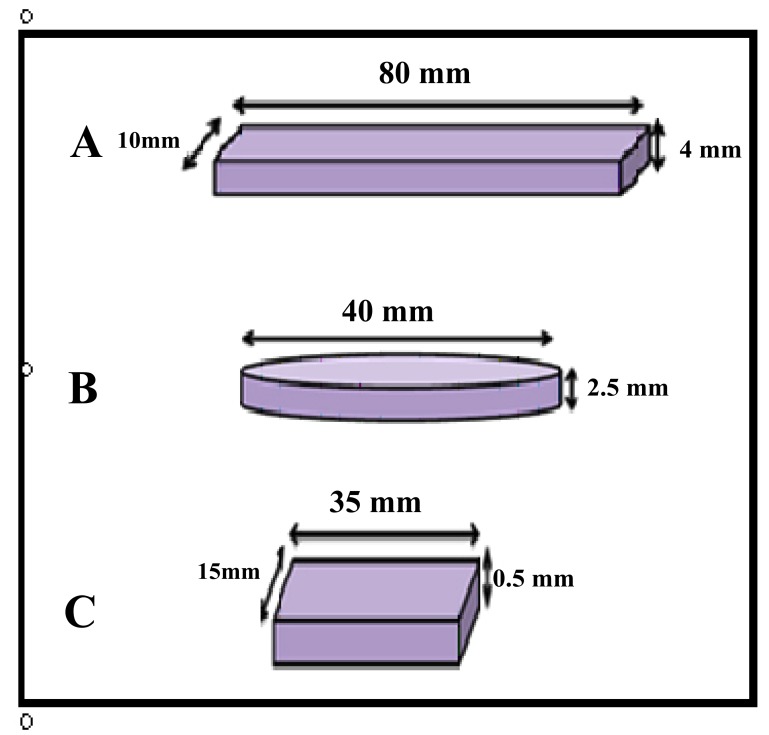
The design and the dimensions for each test,(A) impact strength;(B) thermal conductivity; (C) color stability test.

**Fig. (2):**
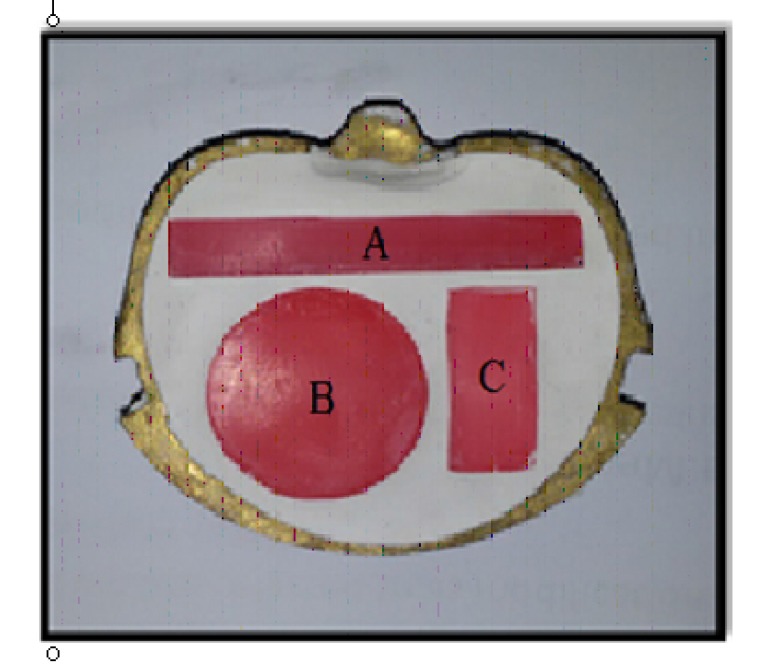
The wax pattern for each test, (A) impact strength; (B) thermal conductivity; (C) color stability test.

The wax patterns (Polywax, Bilkim chemical company, Turkey) were constructed into the desired shape and dimensions for each test (Figs. **1** and **2**) as follows:

1- Impact strength test: a bar-shaped specimen with the dimensions of 80mm x 10mm x 4mm length, width, and thickness respectively [23] according to ISO.179,2000.
2-Color stability test: a rectangular shaped specimen with dimensions of 35mm x 15mm x 0.5mm length, width and thickness respectively aand according to ADA,1999 [24].
3-Thermal conductivity test: disc with dimensions of 40mm in diameter and 2.5mm in thickness according to instrument specification (Thermal constant apparatus).

The mould preparation was utilized by covering the wax pattern with separating medium and left to dry. The mould was made by bagging the lower portion of the flask with dental stone type IV(Elite stone, Zhermack, Germany), that was mixed according to the manufacturer^'^s instructions. The wax patterns were placed into 1/2 of their thickness. After the stone was set, another layer of separating medium was coated and then allowed to dry. After that, the opening of the flask was carefully done and the patterns were removed from the mould**.**

### 2.2 Addition of TiO_2_ Nano-Fillers

**Fig. (3):**
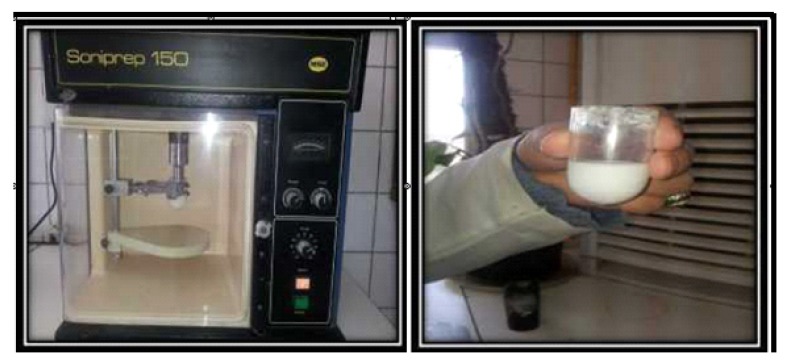
TiO2 Nanofillers were well dispersed in the monomer by a probe sonication apparatus.

The addition of 3% TiO_2_ nanofillers to the monomer was done through the extremely sonication of the fillers which had been well dispersed in the liquid by a probe of sonication equipment (Soniprep150, England) at 120W and 60 KHz to split them into nano-crystals for 3 minutes individually (Fig. **3**) [25]. The monomer with nanoparticles was mixed with acrylic powder immediately to reduce the possibility of particle aggregation and separation. The proportion of acrylic mixing was 2.5:1 (P/L) according to the manufacturer's instruction and left to stand until a dough stage was reached. An electronic balance with 0.0001g accuracy (Staroius BP 30155, Germany) was used to measure the weight of materials that were utilized in this study.

### 2.3 Packing and Curing

The dough stage of the mixture was packed in the mould after lining with a separating medium and become ready for curing. According to the type of curing, the conventional brass metal flask (Broden, Sweden) was used for water bath curing, while for microwave cured the special type of flask fiber reinforced plastic (FRP Flask, G C America) was used.

**Fig. (4):**
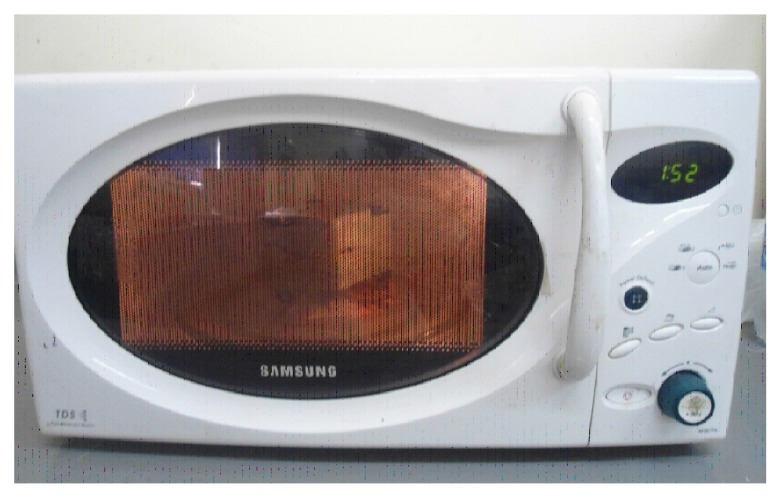
The flask inside the microwave.

There are two methods for curing: the water bath curing which was carried out by placing the clamped flask in the water bath (Memmert, Germany) and processed by heating at 74°C for an hour and a half. The temperature was increased to the boiling point for 30 minutes according to ADA Specification No.12, 1999 [24].The metal flask was allowed to cool at room temperature for 30minutes after completing the curing, followed by complete cooling of the metal flask for 15 minutes before deflasking. Then the specimens were removed from the stone mould. While the microwave curing was carried out by placing the flask in the microwave (Samsung TDS, Korea) and processed by heating at(500W)for 3minutes (Fig. **4**) [26]. After overnight bench cooling, the specimens of microwave-cured acrylic were deflasked [27]. Eventually, finishing and polishing for all samples had been completed.

### 2.4 Testing Procedures

#### 2.4.1 Impact Strength Test

**Fig. (5):**
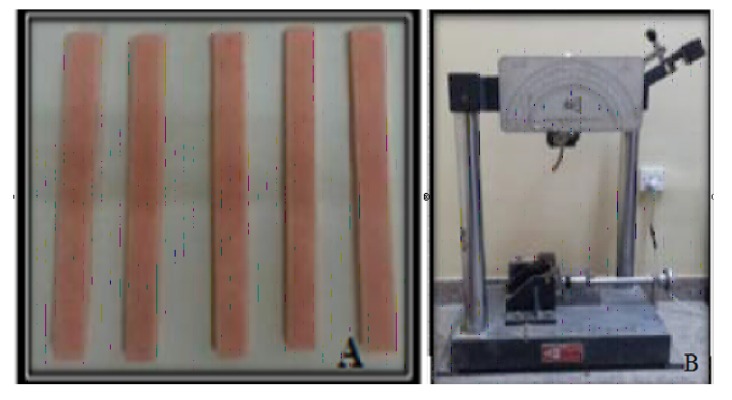
(A) Impact test specimens; (B) Impact strength device.

Acrylic specimens for impact strength test (Fig. **5**) were conditioned by storing in distilled water at 37°C for 48hours before testing [24]. Impact testing device N.43-1, (INC, USA) (Fig. **5**) was used to test impact strength by placing the specimen horizontally where a free-swinging pendulum (2 joules capacity) in the middle wa in a stable height. The reading of the impact energy absorbed to specimen fracture from the scalar was obtained in joules. The “Charpy impact strength” was recorded in KJ/m^2^:

Impact strength= E/ b.d×10^3^ [28]

Where E: is the impact absorbed energy in Joules.

b: is the width in mm of specimens.

d: is the thickness in mm of specimens.

#### 2.4.2 Thermal Conductivity Test

**Fig. (6):**
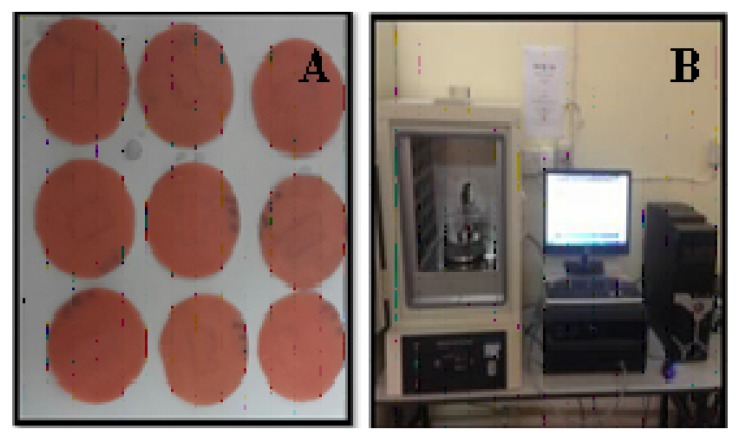
(A)Thermal conductivity specimens;(B) Thermal conductivity test machine.

The disc was made following the specification of the instrument (Thermal constant apparatus) (Fig. **6**).To measure properties of thermal transport, the analyzer hot disc thermal constant was used with thermal conductivities extending from 0.005w/m°c (Evacuated powders) to 500 w/m°c (graphite) [29]. On both sides of the conducting pattern, there was a thin electrically insulating material for supporting. The equipment was connected to computers that were programmed for the test. The experiment was called TPS (transient plane source).

#### 2.4.3 Color Stability Test

**Fig. (7):**
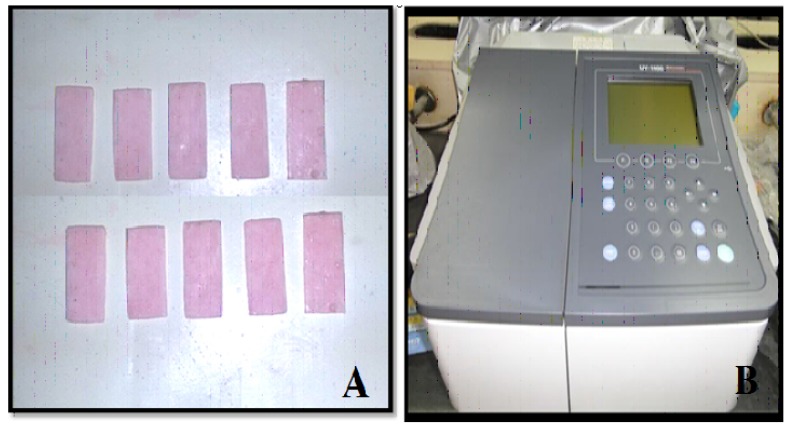
(A) Color stability specimens; (B) Spectrophotometer device.

The spectrophotometer device (Macbeth, USA) was used for measuring the light absorption of each specimen at 500 nm wavelength in order to detect the light surface color absorption, the differences in the light absorption of samples scoring as well as the degree of changes in the color of the specimens between the control and experimental groups [30] (Fig. **7**).

Data were analyzed to conclude the descriptive results as means and standard deviation tables and figures. Student t-test was used to detect the significant differences between tested and control groups at a level of significance (*P*<0.05).

## 3 RESULT

### 3.1 Impact Strength Test

Descriptive statistics of impact strength values showed that the heat-cured had higher mean values than the microwaved-cured samples. The impact strength for those without the addition of 3%TiO_2_ showed a lower mean value than those with an addition of 3%TiO_2_ for both curing methods as shown in Table **1** and Fig. (**8**).

On comparing the effect of the addition of 3%TiO_2_ nano-filler on the impact strength of the heat cured acrylic, the student t-test showed that there was a significant difference between the tested groups (Table **2**).

In comparison, the effect of the curing methods on the impact strength of the heat cured acrylic, the student t-test showed that there was a significant difference in the impact strength between water bath and microwave cured of acrylic resin in the tested groups (Table **3**).

### 3.2 Thermal Conductivity Test

Descriptive statistics of the results of the thermal conductivity values showed that heat-cured specimens without addition of 3%TiO_2_ had lower mean values than the specimens with addition of 3% TiO_2_, and that microwaved-cured specimens with addition of 3% TiO_2_ had higher mean values than those without addition of 3% TiO_2_ asshown in Table **1** and Fig. (**9**).

For comparison of the effect of 3% TiO_2_ addition on the thermal conductivity of the heat-cured acrylic, the student t-test showed that there were non-significant differences between the test groups (Table **2**).

On comparing mean values of the thermal conductivity of the acrylic according to the curing methods, student t-test showed that there were non-significant differences in thermal conductivity between water bath and microwave cured specimens of acrylic resin in the test groups (Table **3**).

## 3.3 Color Stability Test

Descriptive statistics of the color stability value results showed that the heat-cured specimens without addition of 3% TiO_2_ had lower mean values than the specimens with addition of 3% TiO_2_, and that microwaved-cured specimens with addition of 3%TiO_2_ had higher mean values than those without addition of 3% TiO_2_ as shown in Table **1** and Fig. (**10**).

On comparing the effect of the addition of 3% TiO_2_ on the color stability of the acrylic, the student t-test showed that there were significant differences between the tested groups (Table **2**).

Furthermore, depending on the curing methods, the mean values of the color stability of the acrylic resin were compared using student t-test which showed that there were non-significant differences in color stability between water-bath and microwave-cured acrylic resin in the tested groups (Table **3**).

## 4 DISCUSSION

The decision was made to choose the percentage of TiO_2_ nanoparticles about 3% depending on the previous study that was conducted different concentration of TiO_2_ nanoparticles was added to acrylic resin, that was obtained the greatest values of impact strength, flexural strength and surface hardness of heat cured acrylic resin at 3% of nanofiller and these properties decreased with increase of this percentage [20], as well as when the percentage increase above 5% that lead to massive changes occurred in the color of acrylic [31]. On the other hand, higher concentrations (5% TiO_2_) will lead to impact strength deterioration of the resin material, thus causing a decrease in the strength of reinforced specimens that was reported by different authors [31, 32].

Another study reported that the use of 3% of TiO_2_ resulted in antibacterial activity without comprising the mechanical properties and cytotoxicity of PMMA [21]. Another research on the addition of TiO_2_ into PMMA showed that the incorporation of nano-sized TiO_2_ adversely affected the mechanical properties and the flexural strength values with increase in TiO_2_ concentration [33].

The result of the present study revealed that the use of nano-TiO_2_ resulted in higher impact strength, which is attributed to the good bonding between nano-filler and matrix as result of increased interfacial shear strength with decreased crack propagation [34]. The other cause was the very small size of TiO_2_nano-fillers used in this study (about 30 nm) that led to increase in surface area, which was useful for energy dissipation [35]. The use of TiO_2_ also may form a good mesh between the resin and TiO_2_ resulting in slow segmental motion [36]. These results coincided with the research which found that the addition of silanized nano-TiO_2_ increased the value of impact strength [34] and also agreed with the results of the study that showed that the use of 2% nano ZrO_2_: TiO_2_ caused higher impact strength [37].

This study revealed no change in the thermal conductivity by using the TiO_2_, a finding that agreement with the study which concluded that the incorporation of 2% by weight of ZrO_2_: TiO_2_ showed no difference in this property [37], while it disagreed with the study which concluded that the thermal conductivity increased with the use of alumina [29].

For the color changes, the studies have been carried out to confirm and to investigate the color alteration by using apparatus because the human eyes are not sensitive to specific devices [38]. Therefore, color alterations objectively measured the amount of light absorption using a spectrophotometer [39]. The tested acrylic specimens showed a noticeable color change with the addition of TiO_2_. This result in agreed with the study which showed that the increase in light absorption is statistically significant as a result of light absorption with the increasing of modified nano-ZrO_2_ concentration [30]. This is obviously because of the presence of nano-TiO_2_ powder in the matrix which is opaque and absorbs more light energy than polymer matrix and appears more opaque. These findings are attributed to the high atomic number of titanium compared to the low atomic number of the chemical constituent of acrylic which is dependent on the cube of its atomic number [30].

According to the curing methods, the result viewed that there was a significant decrease in the impact of acrylic resin that was cured with microwave, and this may be attributed to the residual monomer that affects the strength of specimens because the low levels of residual monomers increased the strength of resin [40]. Another reason may be that, conventional acrylic resin polymerized in water bath exhibited fewer porosities, unlike the acrylic that was cured by microwave which exhibited a higher change in porosity [26]. The result of this study showed that there was no significant change in thermal conductivity, and this may be due to the microwave heating which is independent of the thermal conductivity. The benefits of curing resin denture base by microwave energy include less cumbersome equipment, greatly reduced curing time and a cleaner method of processing [41]. On the other hand, the result of this study showed that there was no change in the color of the acrylic specimens by microwave curing, which may be due to a shortened dough forming time and more homogeneous resin dough that causes minimal changes in the color of resin base [42].This result is in agreement with Assunçao *et al*., 2009 who found that the color of denture teeth was not affected by the polymerization methods, as there was no significant difference between conventional polymerization and microwave curing [43].

## CONCLUSION

It seems that the curing of high-impact acrylic by microwave had not changed the color stability and thermal conductivity in comparison to the water bath, but it decreased the impact strength. The incorporation of 3% of TiO_2_ improved the impact and the color stability, however, the thermal conductivity was not changed.

## Figures and Tables

**Fig. (8):**
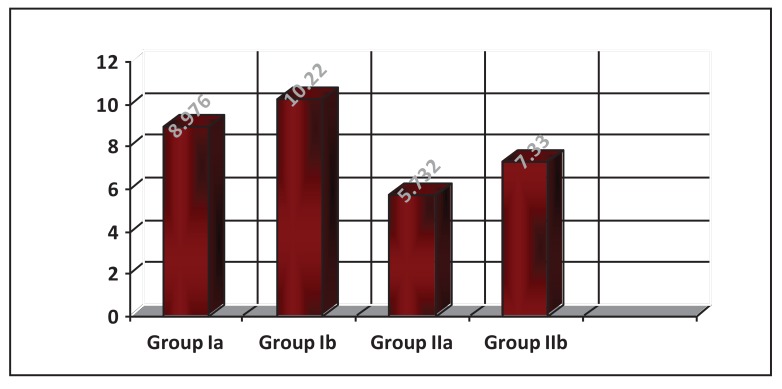
Bar chart showed the Impact strength.

**Fig.(9):**
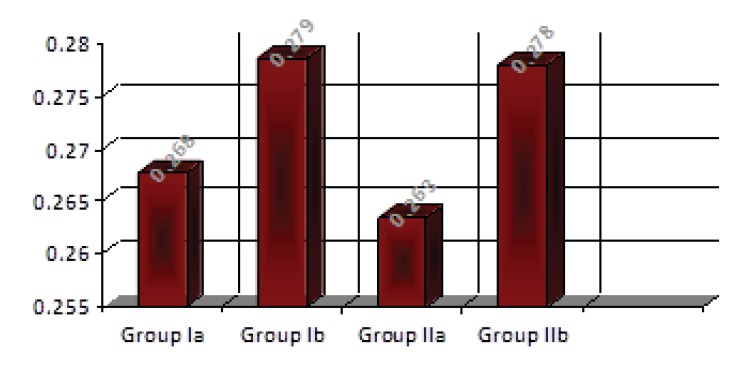
Bar chart showed the Thermal conductivity test.

**Fig. (10):**
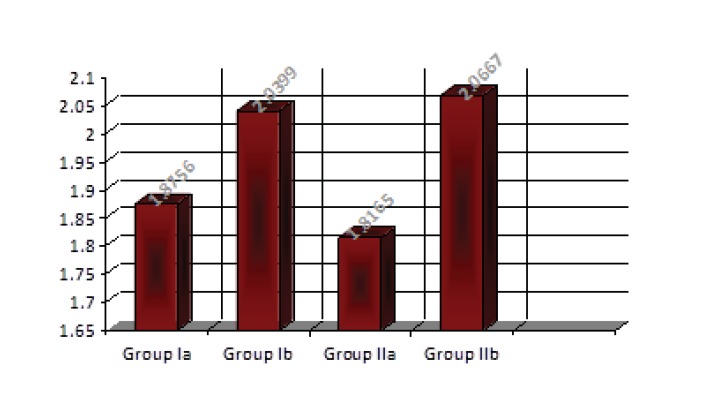
Bar chart showed the color stability test.

**Table 1:** Mean, minimum values, maximum values, and Std. Deviation of Impact strength test, thermal conductivity, and color stability for all groups.

–			**N**	**Mean**	**Min. Value**	**Max. Value**	**Std. Deviation**
**Impact strength**	**Group I** **[water bath]**	**Group Ia: 0%TiO_2_**	10	8.976	8.060	10.060	0.698
		**Group Ib: 3%TiO_2_**	10	10.218	9.190	11.700	0.790
	**GroupII [microwave]**	**Group IIa: 0%TiO_2_**	10	5.732	4.790	6.830	0.605
		**Group IIb: 3%TiO_2_**	10	7.330	6.290	8.790	0.780
**Thermal conductivity**	**Group I** **[water bath]**	**Group Ia: 0%TiO_2_**	10	0.267	0.257	0.278	0.007
		**Group Ib: 3%TiO_2_**	10	0.278	0.231	0.340	0.035
	**GroupII [microwave]**	**Group IIa: 0%TiO_2_**	10	0.263	0.250	0.283	0.0107
		**Group IIb: 3%TiO_2_**	10	0.277	0.251	0.308	0.0199
**Color stability**	**Group I** **[water bath]**	**Group Ia: 0%TiO_2_**	10	1.875	1.770	1.968	0.071
		**Group Ib: 3%TiO_2_**	10	2.039	1.958	2.179	0.074
	**GroupII**	**Group IIa: 0%TiO_2_**	10	1.816	1.667	1.901	0.080
–	**[microwave]**	**Group IIb: 3%TiO_2_**	10	2.066	1.968	2.185	0.086

**Table 2:** T-Test was used for impact strength, thermal conductivity, and color stability between groups according to the addition of 3% of TiO_2_.

–		**t-Test**	***P*-Value**	**Sig**
**Impact strength**	**Group la and Group lb**	3.723	0.002	S
	**Group IIa and Group llb**	5.115	0.000	S
**Thermal conductivity**	**Group la and Group lb**	0.944	0.358	NS
	**Group IIa and Group llb**	2.017	0.059	NS
**Color stability**	**Group la and Group lb**	5.045	0.000	S
	**Group IIa and Group llb**	6.728	0.000	S

**Table 3:** T-Test of impact strength, thermal conductivity, and color stability between groups according to curing methods.

–		**t-Test**	***P*-Value**	**Sig**
**Impact strength**	**Group la and Group lla**	11.098	0.000	S
	**Group lb and Group llb**	8.220	0.000	S
**Thermal conductivity**	**Group la and Group lla**	1.031	0.316	NS
	**Group lb and Group llb**	0.049	0.961	NS
**Color stability**	**Group la and Group lla**	1.741	0.099	NS
	**Group lb and Group llb**	0.746	0.465	NS

*S at *P*-Value (*P* ≤ 0.05).

** NS at *P*-Value (*P*> 0.05).
